# Supplementary material for: Exciton Superposition across Moiré States in a Semiconducting Moiré Superlattice
Source: Nat Commun. 2023 Aug 19;14:5042. doi: 10.1038/s41467-023-40783-z (PMC10439888; doi:10.1038/s41467-023-40783-z)
Supplement: Supplementary file 1 — Supplementary Information [file 41467_2023_40783_MOESM1_ESM.pdf]

Supplementary Information

Exciton Superposition across Moiré States in a Semiconducting Moiré Superlattice

Zhen Lian[1#], Dongxue Chen[1#], Yuze Meng[1], Xiaotong Chen[1], Ying Su[2], Rounak Banarjee[3], Takashi Taniguchi[4], Kenji Watanabe[5], Sefaattin Tongay[3], Chuanwei Zhang[2], Yong-Tao Cui[6*], Su-Fei Shi[1,7*]

1. Department of Chemical and Biological Engineering, Rensselaer Polytechnic Institute, Troy, NY 12180, USA
2. Department of Physics, University of Texas, Dallas, Texas, 75083, USA
3. School for Engineering of Matter, Transport and Energy, Arizona State University, Tempe, AZ 85287, USA
4. International Center for Materials Nanoarchitectonics, National Institute for Materials Science, 1-1 Namiki, Tsukuba 305-0044, Japan
5. Research Center for Functional Materials, National Institute for Materials Science, 1-1 Namiki, Tsukuba 305-0044, Japan
6. Department of Physics and Astronomy, University of California, Riverside, California, 92521, USA
7. Department of Electrical, Computer & Systems Engineering, Rensselaer Polytechnic Institute, Troy, NY 12180, USA

[#] These authors contributed equally to this work
[*] Corresponding authors: shis2@rpi.edu, yongtao.cui@ucr.edu

Summary of contents

Supplementary section 1: Optical images of device D1 and D2

Supplementary section 2: Detailed carrier-density-dependent reflectance contrast of device D1 and D2

Supplementary section 3: Derivative analysis of the reflectance contrast spectra of device D1 and D2

Supplementary section 4: Detailed electric-field-dependent reflectance contrast of device D1 and D2

Supplementary section 5: Polarized second-harmonic generation (SHG) spectra measured from device D1.

Supplementary section 6: Hybridized excitons in another 3L WSe_2/ 1L WS_2 device

Supplementary section 7: Hybridized excitons in natural quadlayer WSe_2 (4L WSe_2)

Supplementary section 8: Electric field dependence of reflectance contrast in natural bilayer WSe_2 (2L WSe_2)

Supplementary section 9: Electric field dependence of reflectance contrast from a natural trilayer WSe_2 region of device D4

Supplementary section 10: Control experiments performed on a 3L WSe_2/ 1L WS_2 device with a large twist angle

Supplementary section 11: Fitting of the reflectance background

Supplementary section 12: Extracted peak positions from Fig. 3a and Fig. 4c

Supplementary section 13: Details of the modeling of hybridized excitons

Supplementary section 14: Discussion of the effect of doping on exciton energies

Supplementary section 15: Electric field dependence of reflectance contrast spectra at different filling factors measured from device D1

Supplementary section 16: Discussion of hole and electron hybridization scenarios

Supplementary section 1: Optical images of device D1 and D2

Fig. S1 shows the optical images of device D1 and D2 shown in the main text.

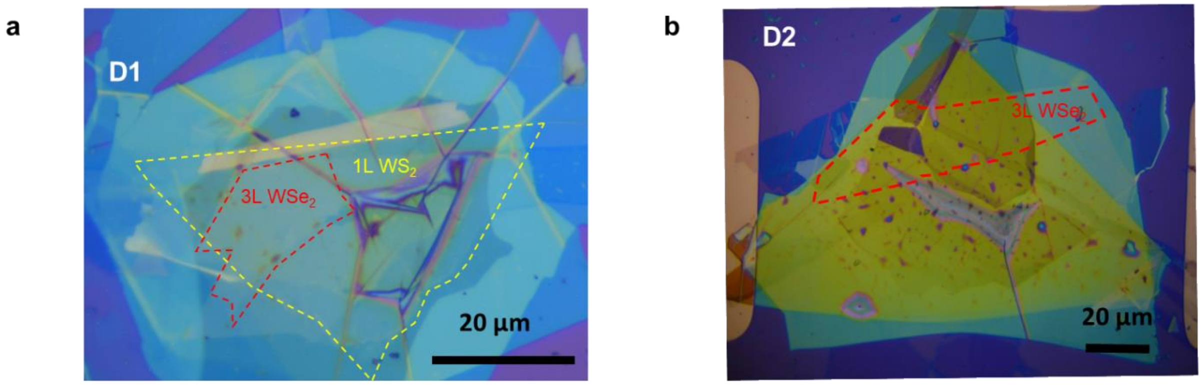

Fig. S1. Schematics and optical images of 3L WSe_2/ 1L WS_2 (D1) and 3L WSe_2 (D2) devices shown in Fig.1. (a) and (b) show the schematic and the optical image of device D1 and D2, respectively.

Supplementary section 2: Detailed carrier-density-dependent reflectance contrast of device D1 and D2

The zoomed-in carrier density dependent reflectance spectra of device D1 and D2 are shown in Fig. S2 to enlarge the high-energy features.

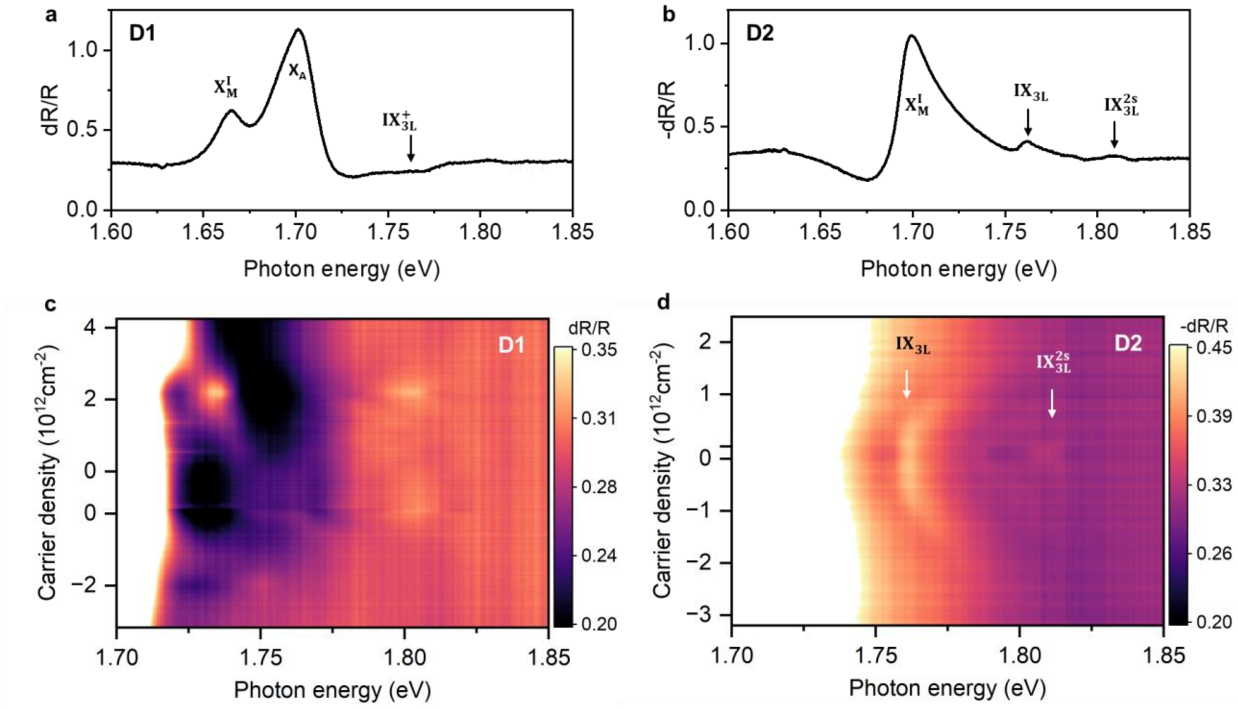

Fig. S2. Detailed reflectance contrast of device D1 and D2. (a) and (b) are the reflectance contrast spectra of device D1 and D2 at charge neutral. (c) and (d) are the zoomed-in gate dependent reflectance contrast spectra of Fig. 2e (c) and Fig. 2f (d).

Supplementary section 3: Derivative analysis of the reflectance contrast spectra of device D1 and D2

To manifest the subtle features on the gate dependence of the reflectance spectra, we take the derivatives of the dR/R with respect to carrier density or electric field, as shown in Fig. S3. Fig. 3a and c are the derivatives of dR/R with respect to carrier density from device D2 and D1, respectively. The features corresponding to the correlated insulating states at integer fillings of the moiré superlattice are clearly visible in Fig. S3c. Figs. 3b and d are the derivatives of dR/R with respect to electric field from device D2 and D1, respectively. It is clear that device D1 (3L WSe_2/ 1L WS_2) shows a three-level hybridization in Fig. S3d, in sharp contrast to the two-level hybridization from device D2 (3L WSe_2) shown in Fig. S3b.

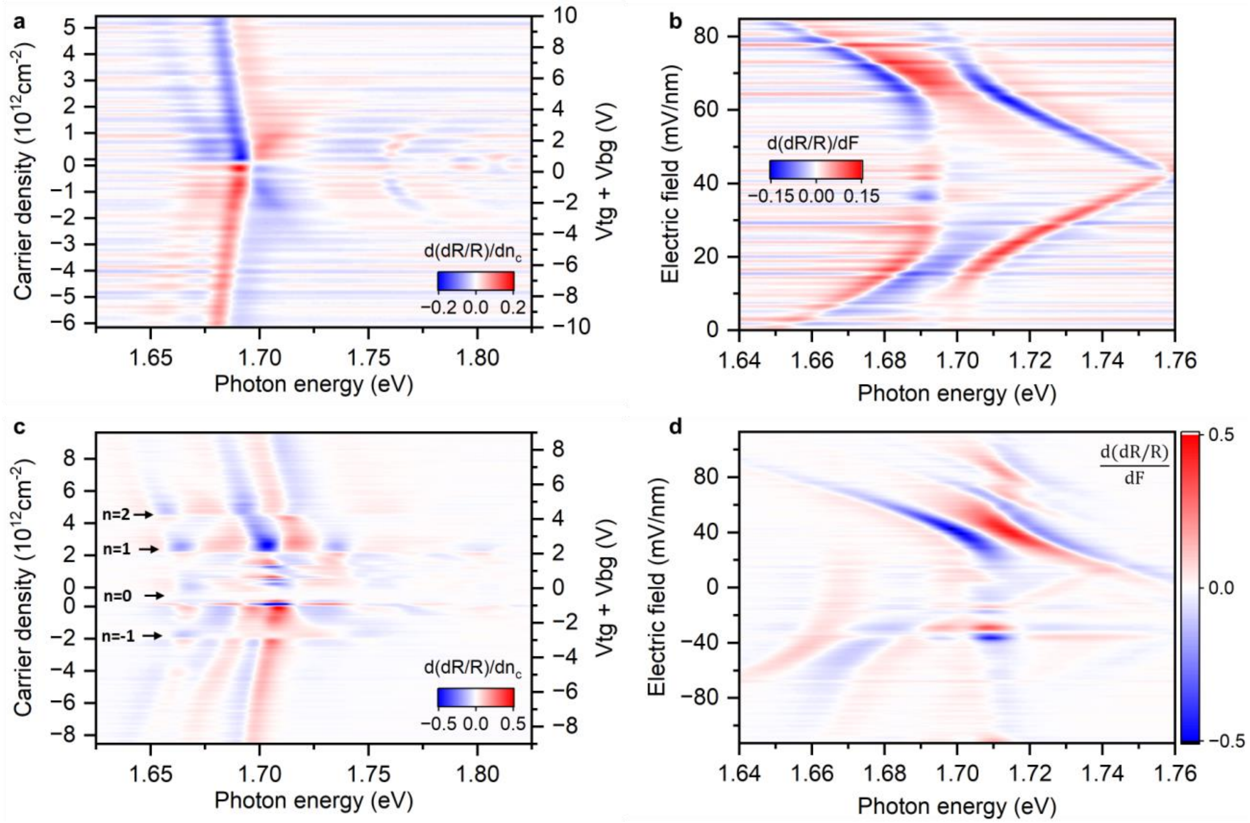

Fig. S3. Derivative analysis of the reflectance contrast spectra of device D1 and D2. (a) and (c) show the derivative of dR/R from Fig. 2 f and e with respect to carrier density, respectively. (b) and (d) show the derivative of dR/R from Fig.3a and Fig.4c with respect to electric field, respectively.

Supplementary section 4: Detailed electric-field-dependent reflectance contrast of device D1 and D2

The zoomed-in electric field dependent reflectance spectra of device D1 and D2 are shown in Fig. S4 to enlarge the high-energy features.

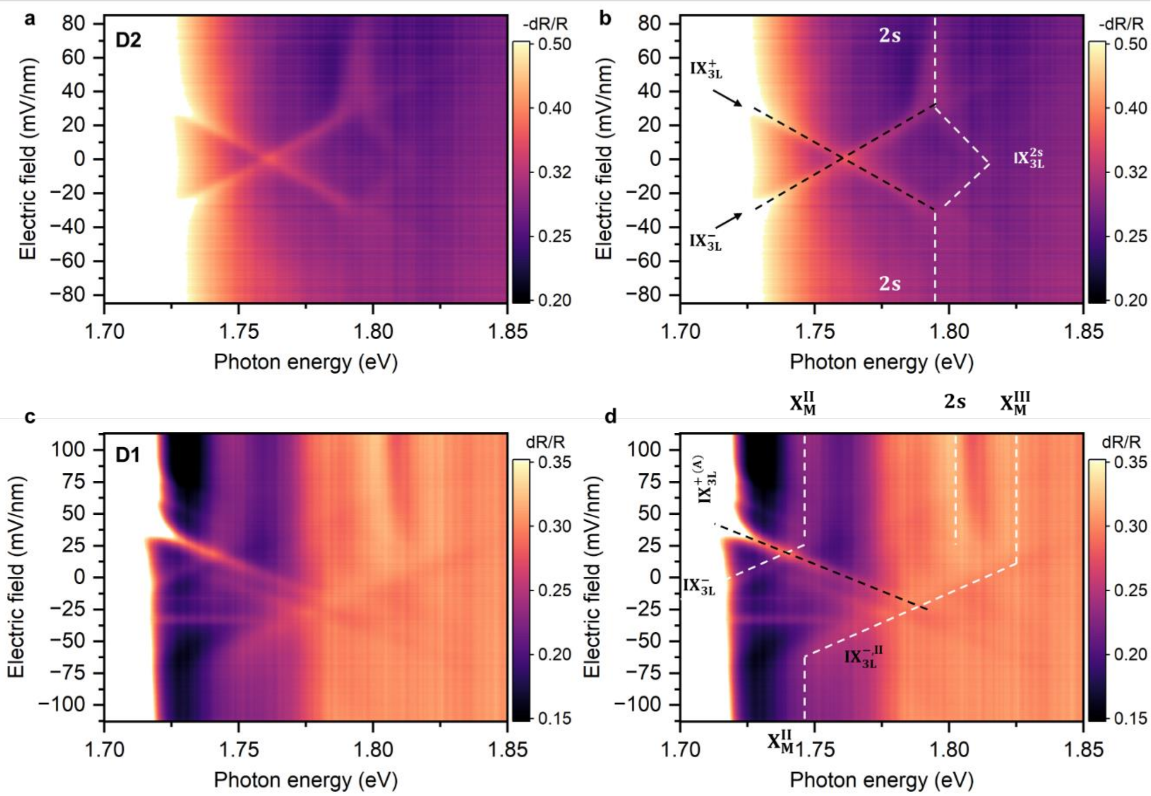

Fig. S4. Reflectance contrast spectra of zoom-in regions of Fig. 3a (a, b) and Fig. 4a (c, d). (b) and (d) label the features in (a) and (c) with dashed lines as eye guides, respectively.

Supplementary section 5: Polarized second-harmonic generation (SHG) spectra measured from device D1.

Fig. S5a shows the integrated SHG intensity as a function of polarization angle measured from the 3L WSe_2 region and the monolayer WS_2 region of device D1. The SHG spectra from the 3L WSe_2 region, the monolayer WS_2 region and the 3L WSe_2/ 1L WS_2 heterostructure on device D1 are shown in Fig. S5b.

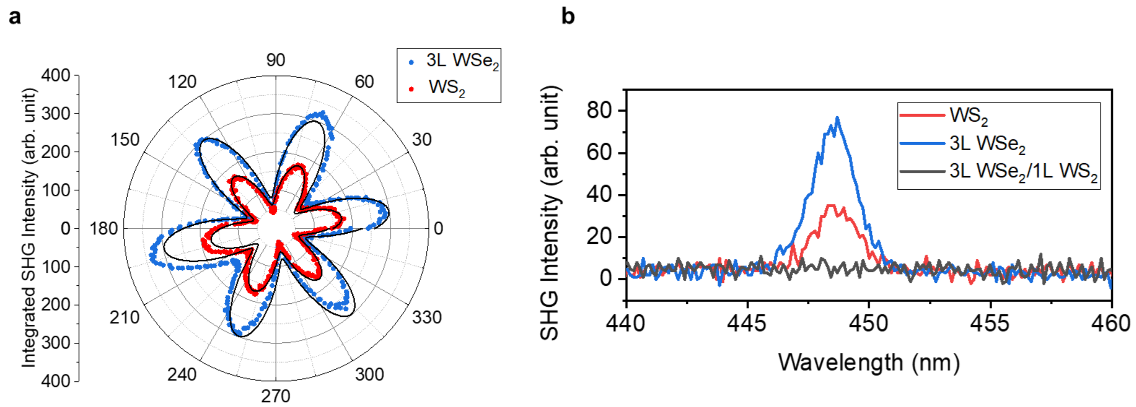

Fig. S5. Second-harmonic generation (SHG) spectra as a function of polarization angle from 3L WSe_2/ 1L WS_2 device D1. (a) shows the integrated SHG intensity as a function of polarization angle from the 3L WSe_2 region and 1L WS_2 region. (b) shows the SHG spectra from 3L WSe_2, 1L WS_2 and 3L WSe_2/ 1L WS_2 heterostructure. The quench of the SHG signal on 3L WSe_2/ 1L WS_2 indicates the alignment angle is close to 60°. By fitting the polarization angle dependence of the SHG signal with a sinusoidal function, we determine the twist angle to be 0.9° ± 0.5°.

Supplementary section 6: Hybridized excitons in another 3L WSe_2/ 1L WS_2 device

Fig. S6 shows the reflectance contrast spectra and the derivative of reflectance contrast with respect to the electric field of another 3L WSe_2/ 1L WS_2 device (D3).

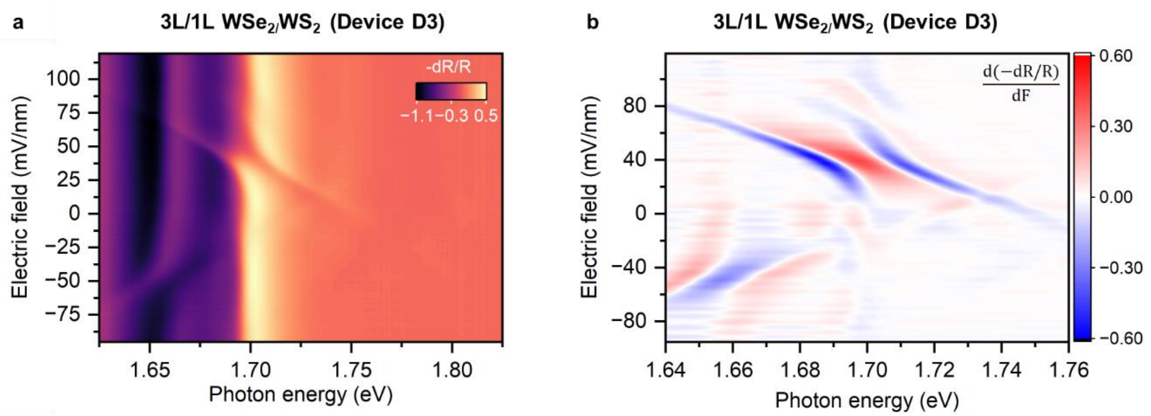

Fig. S6. Reflectance contrast spectra and the derivative of reflectance contrast with respect to the electric field of another 3L WSe_2/ 1L WS_2 device (D3). (a) and (b) show dR/R and d(dR/R)/dF measured from 3L WSe_2/ 1L WS_2 device D3.

Supplementary section 7: Hybridized excitons in natural quadlayer WSe_2 (4L WSe_2)

Fig. S7a shows the reflectance contrast spectra as a function of electric field measured from the 4L WSe_2 region of device D4. Fig. S7b shows the zoom-in of the high energy features in Fig. S7a. Figs. S7c and d are the derivatives of the reflectance contrast with respect to photon energy, d(dR/R)/d(hv), corresponding to Figs. S7a and b, respectively.

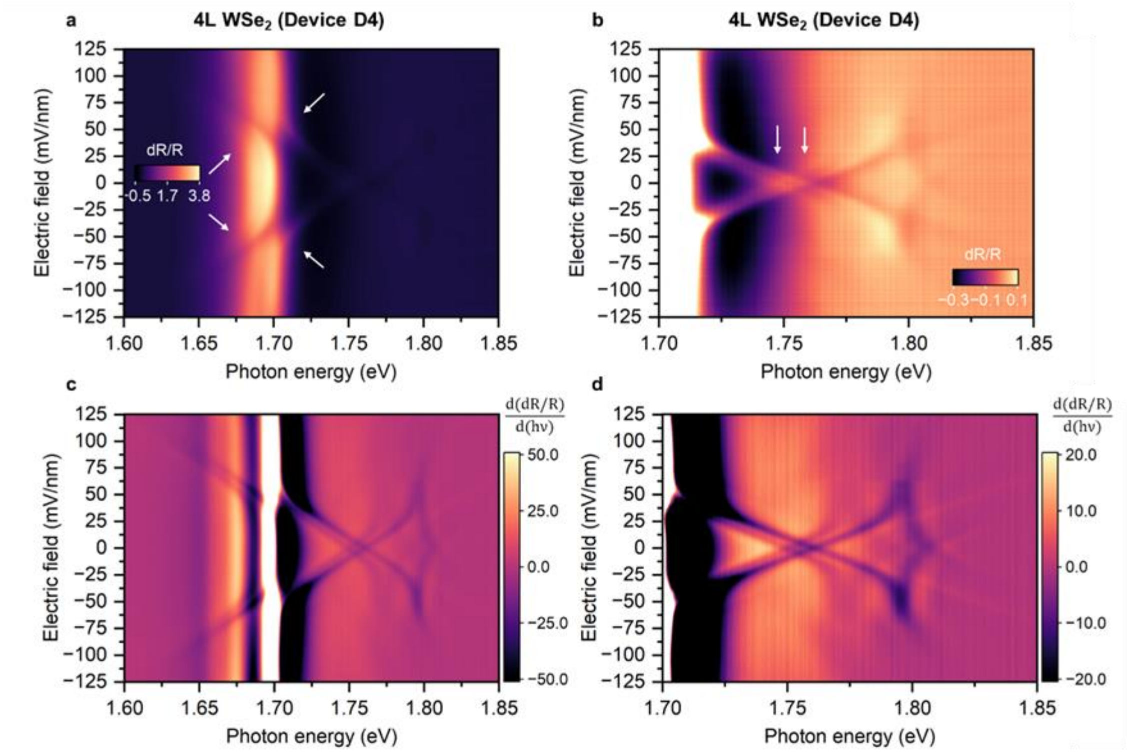

Fig. S7. Electric-field-dependent reflectance contrast spectra of dual gated natural 4L WSe_2 regions of device D4. (a) and (b) are spectra from the 4L region. (c) and (d) are derivatives of reflectance contrast with respect to photon energy corresponding to (a) and (b), respectively.

Supplementary section 8: Electric field dependence of reflectance contrast in natural bilayer WSe_2 (2L WSe_2)

Fig.S8 shows the electric field dependence of the reflectance contrast spectra measured from a natural bilayer region of device D4. The interlayer hybridized excitons observed in device D2 are absent from Fig.S8.

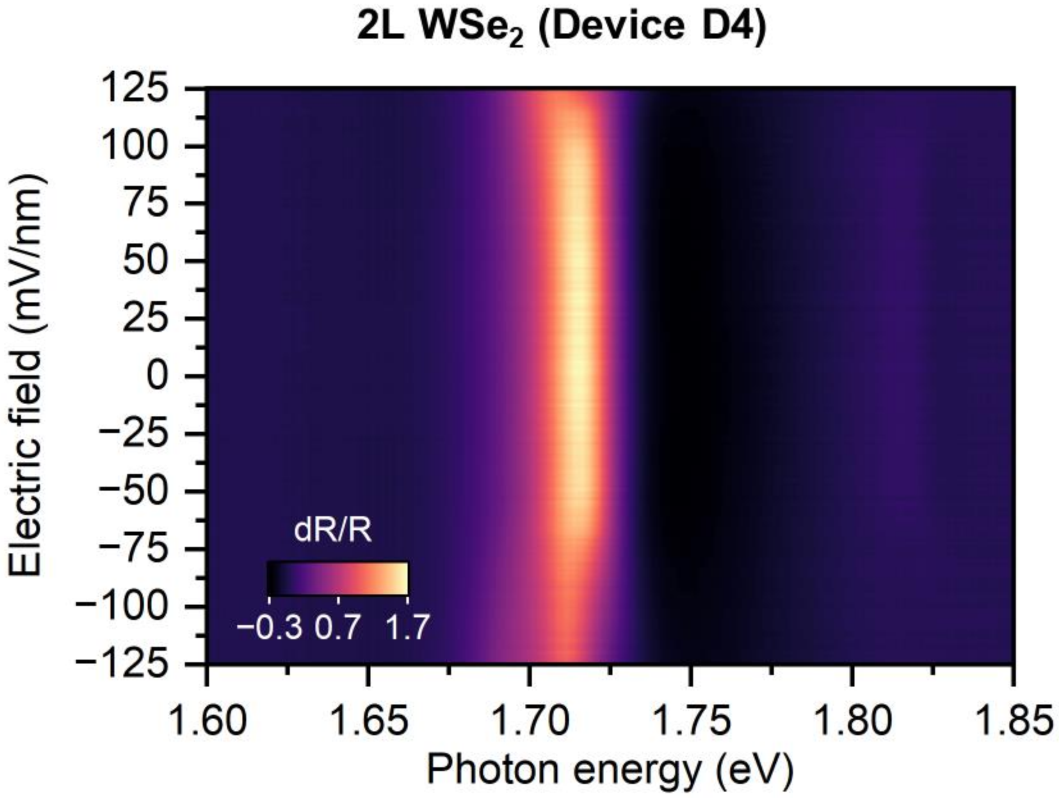

Fig. S8. Electric-field-dependent reflectance contrast spectra from the dual-gated 2H bilayer WSe_2 region of device D4.

Supplementary section 9: Electric field dependence of reflectance contrast from a natural trilayer WSe_2 region of device D4

The electric field dependence of reflectance contrast from the 3L WSe_2 region of device D4 is shown in Fig.S9.

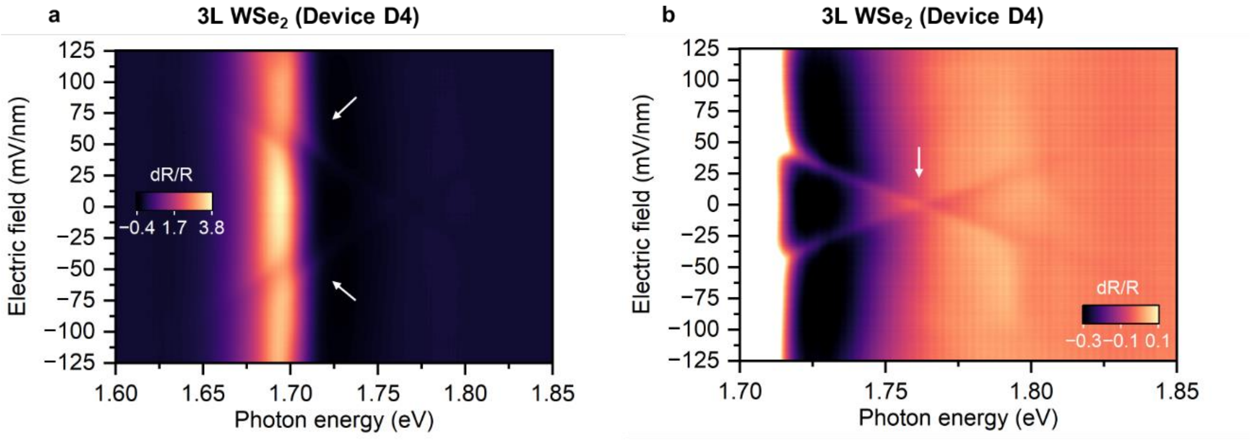

Fig. S9. Electric-field-dependent reflectance contrast spectra of dual gated natural 3L WSe_2 regions of device D4. (a) and (b) are spectra from the 3L region.

Supplementary section 10: Control experiments performed on a 3L WSe_2/ 1L WS_2 device with a large twist angle

Fig. S10a shows the reflectance contrast spectrum measured from a 20°-aligned 3L WSe_2/ 1L WS_2 region of device D4 at zero electric field. For the purpose of comparison, we also replotted the reflectance contrast spectrum from device D1 (Fig. S2a) as Fig. S10b. The exciton resonance X_M^I is absent from Fig. S10a, suggesting it is an intralayer moiré exciton.

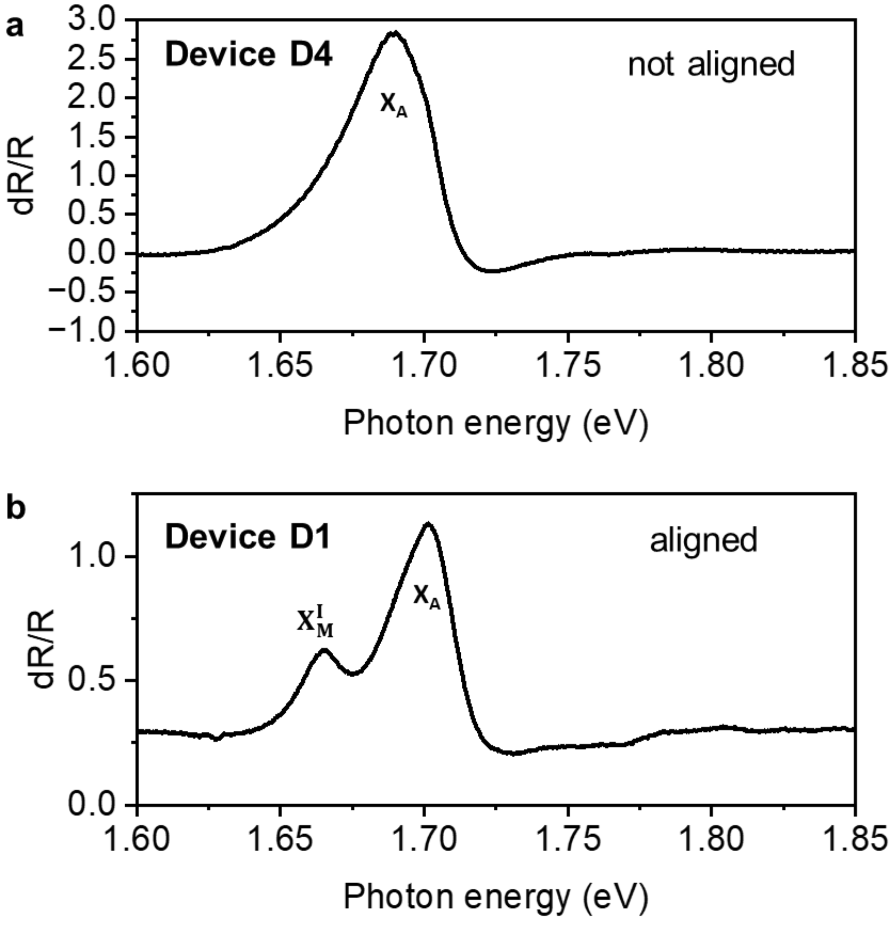

Fig. S10. Comparison of the reflectance contrast spectra from 20°-aligned and close-to-60°-aligned 3L WSe_2/ 1L WS_2. (a) shows the reflectance spectrum at zero electric field measured from the 20°-aligned 3L WSe_2/ 1L WS_2 region of device D4. (b) shows the data from the same measurement on device D1. The data in (b) is the same data from Fig. S2a.

Fig. S11a shows the electric field dependence of the reflectance contrast spectra measured from a 20°-aligned 3L WSe_2/ 1L WS_2 region of device D4. Fig. S11b shows the zoom-in of the high energy features in Fig. S11a. Compared to the reflectance measured from device D1, the splitting of interlayer exciton IX_{3L}^{+} is not observed, suggesting it is related to the moiré effect.

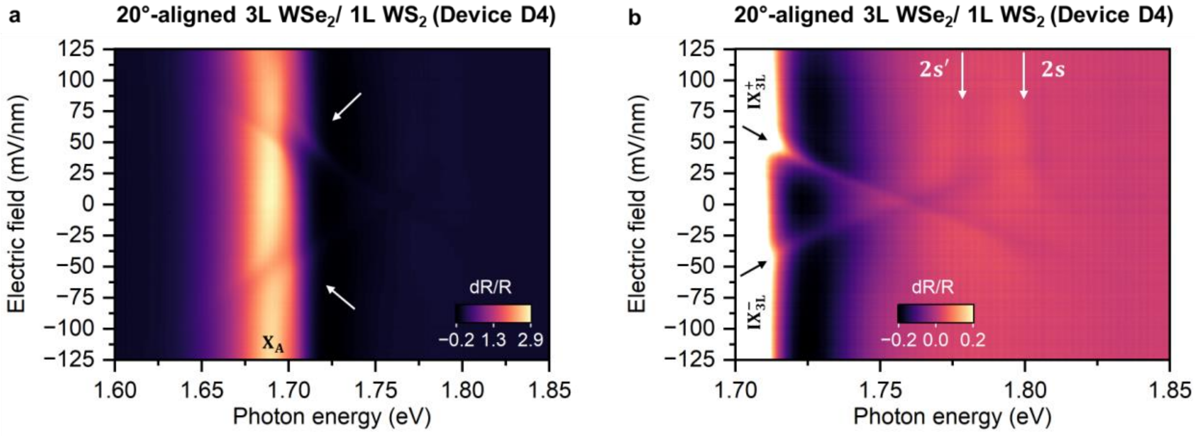

Fig. S11. Reflectance contrast spectra of a dual-gated 3L WSe_2/ 1L WS_2 device with a 20-degree twist angle. (a) and (b) are the electric-field-dependent spectra for different photon energy ranges. The moiré exciton $\mathrm{X}_{\mathrm{M}}^{\mathrm{I}}$ is absent in (a), and the hybridization of interlayer exciton $\mathrm{X}_{3L}^{\mathrm{I}}$ is symmetric about electric field in both (a) and (b).

Supplementary section 11: Fitting of the reflectance background

We use the reflectance contrast spectrum R_h at high hole-doping level ($> 6 \times 10^{12}\ cm^{-2}$) to construct the reflectance background for each spot measured. The high-energy features on the reflectance spectra, including moiré excitons and excited states, disappear due to the screening effect at such a doping level. The features corresponding to exciton resonance X_M^I, $X_A^{'}$ and X_A are identified by comparing R_h with the reflectance spectra from regions with only h-BN and are then removed from the background. The rest of the spectrum is fitted by a polynomial function to construct a flat reflectance background R_0.

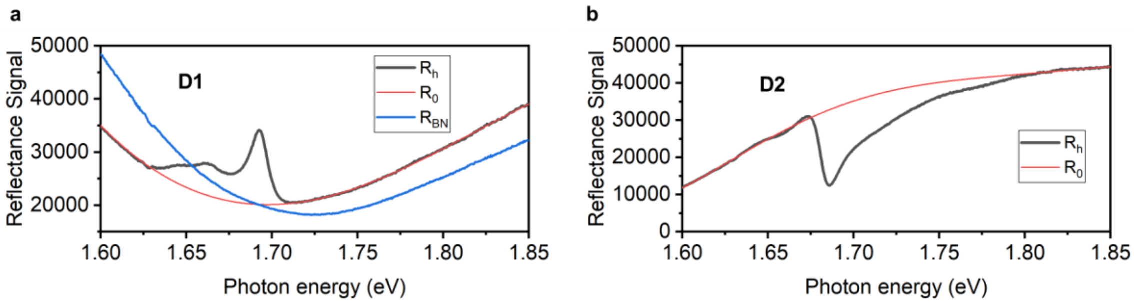

Fig. S12 Fitting of the reflectance background of device D1 and device D2. (a) shows the reflectance spectrum measured on 3L/1L WSe_2/WS_2 at a high doping level, the reflectance spectrum measured on h-BN, and the fitted reflectance background of device D1. (b) shows the reflectance spectrum measured on 3L WSe_2 and the fitted reflectance background of device D2.

Supplementary section 12: Extracted peak positions from Fig. 3a and Fig. 4c

Fig. S13 shows the extracted peak positions from Fig. 4c and Fig. 3a of the main text, which is further used to fit the hybridized exciton models described in supplementary section 13.

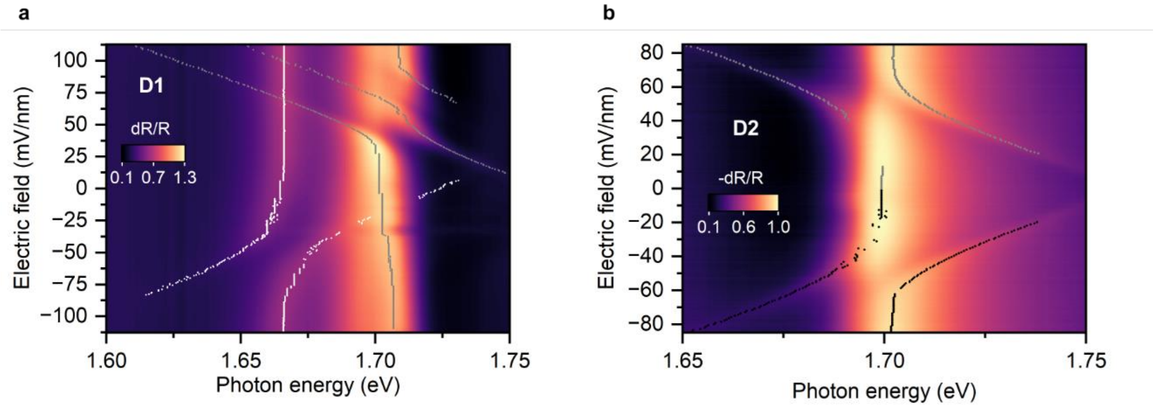

Fig. S13. Extracted peak positions from the electric field dependence of reflectance contrast spectra of devices D1 and D2. (a) and (b) are the extracted peak positions overlayed on the colorplot of Fig. 4c and Fig. 3a. The dots indicate the fitted peak positions.

Supplementary section 13: Details of the Modeling of hybridized excitons

We model the intralayer exciton energy as a constant under a vertical electric field, which is denoted as Xa. The interlayer exciton energy can be expressed as $Xi(F) = Xi0 - e \cdot d \cdot F$, where Xi0 is the interlayer exciton energy at 0 electric field, e is the elementary charge, *d* is the interlayer distance (defined as positive when the dipole moment is downward and negative when upward) and *F* is the vertical electric field inside the heterostructure. In Figs. 3b, 3c and 4d, the hybridized exciton can be modeled by considering the hybridization between one intralayer exciton and one interlayer exciton, which is given by the following Hamiltonian:

$$\begin{bmatrix} Xa & \Delta 1 \\ \Delta 1 & Xi1 - e \cdot d \cdot F \end{bmatrix} \quad (S1)$$

where Δ is the strength of hybridization, and it is a constant in our model.

In Fig. 4e, there are two interlayer excitons involved in the hybridization, and the Hamiltonian is given by:

$$\begin{bmatrix} Xa & \Delta 1 & \Delta 2 \\ \Delta 1 & Xi1 - e \cdot d1 \cdot F & 0 \\ \Delta 2 & 0 & Xi2 - e \cdot d2 \cdot F \end{bmatrix} \quad (S2)$$

The eigenvalues ξ_j of the Hamiltonian give the energies of the hybridized exciton branches.

The extracted peak positions are fitted using F as an independent variable, ξ_j as a dependent variable, and Xa, $Xi0$, Δ and *d* as unknown parameters. A gradient search approach is used to minimize the mean squared error, defined as $MSE = \frac{1}{N}\sum_F \sum_j (y_j(F) - \xi_j(F))^2$. The fitting results from devices D1 and D2 are listed in Table S1.

Device	D2		D1	
Hybridized exciton	Xh(-)	Xh(+)	Xh(-)	Xh(+)
Xa (eV)	1.7001±0.0001	1.6999±0.0001	1.6668±0.0001	1.7071±0.0001
Xi1 (eV)	1.7601±0.0001	1.7603±0.0003	1.7200±0.0001	1.7601±0.0002
Xi2 (eV)	--	--	--	1.8293±0.0003
d1 (nm)	-1.262±0.003	1.262±0.008	-1.231±0.003	1.313±0.004
d2 (nm)	--	--	--	1.609±0.004
Δ1 (meV)	10.1±0.1	10.7±0.3	11.4±0.1	11.1±0.2
Δ2 (meV)	--	--	--	10.9±0.2

Table S1. Summary of the hybridized exciton model parameters extracted from fitting.

Supplementary section 14: Discussion of the effect of doping on exciton energies

Adopting the dielectric constants of 3.5 for hBN, 6.3 for monolayer WS_2, and 7.5 for monolayer WSe_2[1], in the natural 3L WSe_2 device, the second WSe_2 layer has a higher average permittivity environment comparing to the third WSe_2 layer considering the configuration of different WSe_2 layers. The higher dielectric constant can tune more bandgap and binding energy and will induce a corresponding larger energy redshift of intralayer exciton[2,3,] so we attributed the two branches labeled as X_A and X_A' in Fig. 2f to the intralayer exciton in the first/third and second layer WSe_2, respectively.

As both X_A and X_A' redshift linearly with increasing doping under no matter hole or electron doping, we calculated the slopes of doping-dependent energy shifts shown in Table S2, which represent the sensitivity of the intralayer exciton energy to the doping level. The slopes at n doping and p doping for X_A and X_A' are similar, and the slope of X_A' is larger than X_A's. In addition, the dielectric constant of the doped layers will increase because of the accumulation of free carriers. Correspondingly, this overall increase of permittivity environment will lead to a doping-dependent redshift of both X_A' and X_A. However, due to the configuration difference between the second and third WSe_2 layer; that is both doped first/third WSe_2 layers adjacent to the second WSe_2 layer but the two doped first/second (second/third) WSe_2 layers on the bottom (top) side of the third (first) WSe_2 layer, doping will induce a more significant average dielectric environment modification for the second WSe_2 layer than the first/third WSe_2 layer which accounts for a more sensitive doping dependent energy redshift of the intralayer exciton in second WSe_2 layer.

Analogous to the discussion of natural trilayer WSe_2, for the 3L/1L device, we conclude that X_A originates from the third layer WSe_2 and X_A' from the second layer WSe_2. The same local dielectric screening mechanism as natural 3L WSe_2 results in the doping-dependent redshift of X_A and X_A' at the n doping region. At the p doping region, the larger doping sensitivity can be explained by the fact that the second WSe_2 layer is closer to n-doped WS_2 than the third WSe_2. In addition, the energy jump of X_A between doping levels n=1 and n=2 results from the doping dependent hybridization between interlayer exciton and intralayer exciton, which is discussed in Fig. S14.

3L	X_A' (p doping)	X_A (p doping)	X_A' (n doping)	X_A (n doping)
Energy shift/doping (meV/ $10^{12}cm^{-2}$)	2.80	1.26	2.66	1.35
3L/1L	X_A' (p doping)	X_A (p doping)	X_A' (n doping)	X_A (n doping)
Energy shift/doping (meV/ $10^{12}cm^{-2}$)	2.06	1.01	2.05	1.04

Table S2. Summary of the energy shifts of X_A' and X_A as functions of carrier density.

It is worth noting that although the slopes for X_A' and X_A are different for the natural trilayer (3L) WSe_2 device and the 3L/1L WSe_2/WS_2 moiré heterojunction device, likely due to dielectric environment difference, the ratio of the slope of X_A' to that of X_A is about 2.0, similar to that of natural trilayer WSe_2 (2.2 for p-doping and 2.0 for n-doping). This further confirms that the nature of X_A' and X_A observed in 3L/1L WSe_2/WS_2 moiré heterojunction is the same as that of natural trilayer WSe_2.

Supplementary section 15: Electric field dependence of reflectance contrast spectra at different filling factors measured from device D1

At different filling factor n (number of electrons or holes per moiré supercell, “+” for electrons and “-” for holes), we studied the electric field dependent reflectance contrast spectra of 3L/1L WSe_2/WS_2 device (D1, shown in the main text) in Fig. S14. It is evident that as the electron doping increases, the hybridization electric field for the two interlayer excitons and intralayer exciton shifts to a smaller magnitude, approaching zero for n=2. This is because the electrostatically introduced electrons go to the WS_2 layer due to the type II alignment. As a result, the WSe_2 layers will experience an effective electric field pointing towards the WS_2 layer same direction as the positive electric field needed to realize the hybridization of the interlayer moiré excitons with intralayer excitons in the third WSe_2 layer.

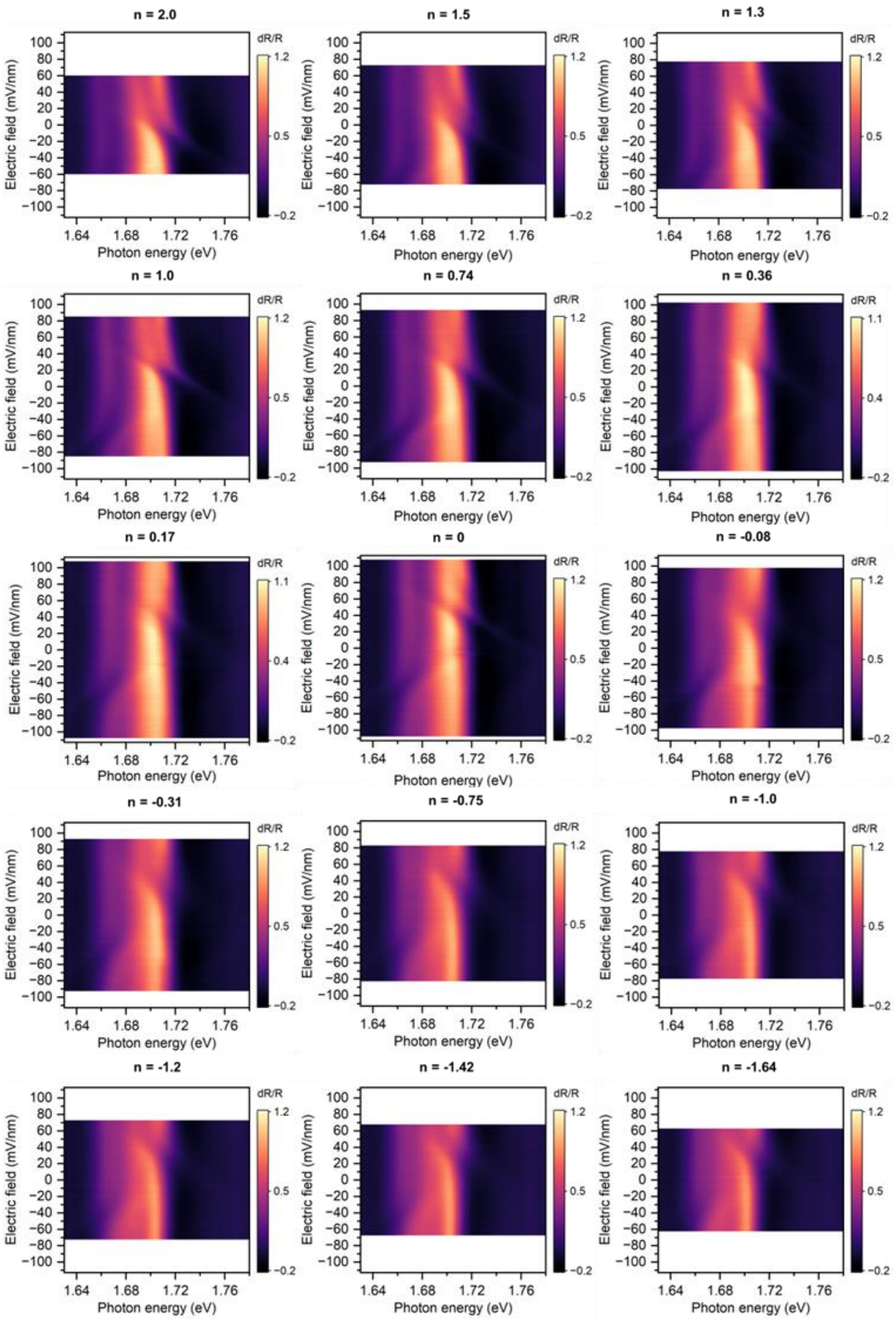

Fig.S14. Electric field dependence of reflectance contrast spectra at different filling factors measured from device D1.

Supplementary section 16: Discussion of hole and electron hybridization scenarios

Considering the upward and downward interlayer exciton dipoles, along with the possibilities of electron or hole tunneling, the different hybridization scenarios are schematically illustrated in Fig. S15a, b, d, e. Fig. S15b and Fig. S15d correspond to the scenarios of hole tunneling, which have been discussed in the main text. Fig. S15a and Fig. S15e correspond to the hybridization of $\mathrm{IX}_{3\mathrm{L}}^{+}$ with X_M^I, and the hybridization of $\mathrm{IX}_{3\mathrm{L}}^{-}$ with $\mathrm{X_A}$ when electron tunneling is considered. It is clear from Fig. S15c that associated excitons cross each other without level avoiding (white arrows marked by ‘a’ and ‘e’), confirming the validity of our discussion in the main text that ignore the conduction band hybridization.

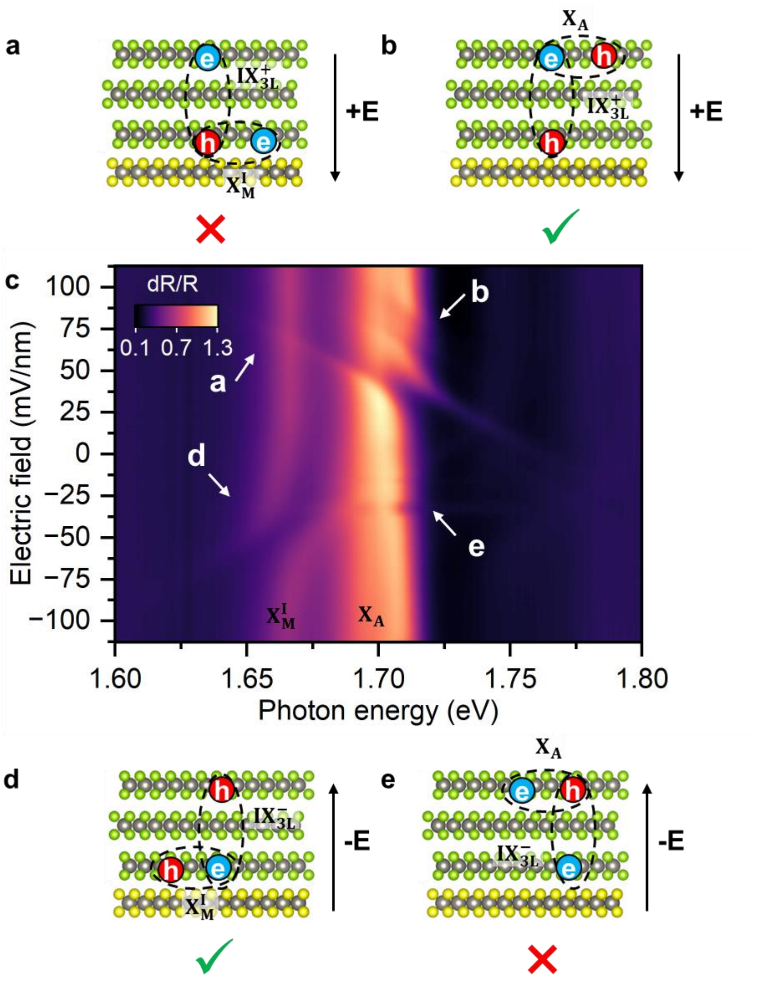

Fig.S15. Different hybridization scenarios in 3L/1L WSe_2/WS_2. (a) and (b) are the schematics showing the hybridization of intralayer exciton with interlayer exciton IX_{3L}^{+}. (d) and (e) are the schematics showing the hybridization of interlayer excitons with interlayer exciton IX_{3L}^{-}. (c) shows the same data as in Fig. 3a, with white arrows indicating the positions where hybridization can possibly occur, considering both electron and hole tunneling.

References

1. Laturia, A., van de Put, M. L. & Vandenberghe, W. G. Dielectric properties of hexagonal boron nitride and transition metal dichalcogenides: from monolayer to bulk. *NPJ 2D Mater. Appl.* **2**, 6 (2018).
2. Raja, A. *et al.* Coulomb engineering of the bandgap and excitons in two-dimensional materials. *Nat. Commun.* **8**, 15251 (2017).
3. Feng, S. *et al.* Highly tunable ground and excited state excitonic dipoles in multilayer 2H-$MoSe_2$. Preprint at https://doi.org/10.48550/ARXIV.2212.14338 (2022).
